# Trends in Bone Marrow Donor Registration in Japan Following a Celebrity’s Disclosure of Leukemia

**DOI:** 10.1001/jamanetworkopen.2021.18698

**Published:** 2021-07-29

**Authors:** Takashi Yoshioka, Takahiro Tabuchi, Yohei Maeda, Yusuke Tsugawa

**Affiliations:** 1 Center for Innovative Research for Communities and Clinical Excellence (CiRC^2^LE), Fukushima Medical University, Fukushima, Japan; 2 Cancer Control Center, Osaka International Cancer Institute, Osaka, Japan; 3 Department of Otorhinolaryngology–Head and Neck Surgery, Osaka University Graduate School of Medicine, Osaka, Japan; 4 Division of General Internal Medicine and Health Services Research, David Geffen School of Medicine at UCLA, Los Angeles, California; 5 Department of Health Policy and Management, UCLA Fielding School of Public Health, Los Angeles, California

## Abstract

This cohort study examines the association of a celebrity’s public disclosure of an acute lymphocytic leukemia diagnosis and trends in bone marrow donor registration in Japan.

## Introduction

The demand for bone marrow far exceeds the global supply, resulting in many patients with hematological diseases or immunodeficiency to wait years or even die while waiting for donated bone marrow.^1^ Increasing the number of bone marrow donor registrations (BMDRs) is one way to address the shortage of bone marrow; however, little is known about effective interventions to increase BMDRs. Given that Angelina Jolie’s *New York Times* editorial increased *BRCA* gene testing during 2012 and 2013 in the United States,^2^ it is also possible that similar media coverage about celebrities’ announcements regarding their (or their family member’s) leukemia may increase BMDRs. On February 12, 2019, a famous swimmer, Rikako Ikee, disclosed her acute lymphocytic leukemia diagnosis via Twitter, which was covered widely by the media in Japan.^3,4^ We examined its association with BMDR.

## Methods

This study was deemed exempt from review by the Fukushima Medical University institutional review board based on the Japanese ethical guideline regarding publicly available data. We followed the Strengthening the Reporting of Observational Studies in Epidemiology (STROBE) reporting guideline for cohort studies.

Using data from the Japan Marrow Donor Program, which exhaustively obtains the number of BMDRs throughout Japan,^5^ we collected the monthly number of BMDRs between September 2015 and August 2019. We classified the monthly reports into years (September of the year through August of the following year).

To estimate the association of Ms Ikee’s disclosure with BMDR, we used an event study design,^6^ and compared the change in the number of BMDRs in the event year (the event date was February 12, 2019; in the period between September 2018 and August 2019) with 3 previous years (between 2015-2016, 2016-2017, and 2017-2018) as the control years. We fit a multivariable ordinary least-squares regression model to the outcome (ie, the monthly number of BMDRs). We regressed the log-transformed number of new BMDRs on indicator variables for time (reference category was defined as January [1 month before the event]),^6^ a binary categorical variable for intervention year group (2018-2019 was defined as intervention), the interaction term between these 2 regressors, and the indicator variables for the year.

Statistical significance was set at *P* < .05, and testing was 2-sided. The data were analyzed using STATA version 16.1 (StataCorp) from February to April 2021.

## Results

Our sample included 155 948 BMDRs, of which 56 989 (36.5%) were registered in 2018-2019. The yearly BMDR trends were similar except for the intervention year (Figure). There were more monthly BMDRs after Ms Ikee’s disclosure than the same month (February) in the previous years (2016: 2284 cases; 2017: 2437 cases; 2018: 2570 cases; and 2019: 11 662 cases). The event study analysis showed that Ms Ikee’s disclosure was associated with the increase in BMDR (adjusted difference, +348%; 95% CI, +318% to +379%; *P* < .001). The increasing trends attenuated over time, but remained higher than usual for at least the next 7 months (until the end of the study period) (Table). We found no evidence that the number of bone marrow transplantations performed increased following Ms Ikee’s disclosure (data not shown).

**Figure.**
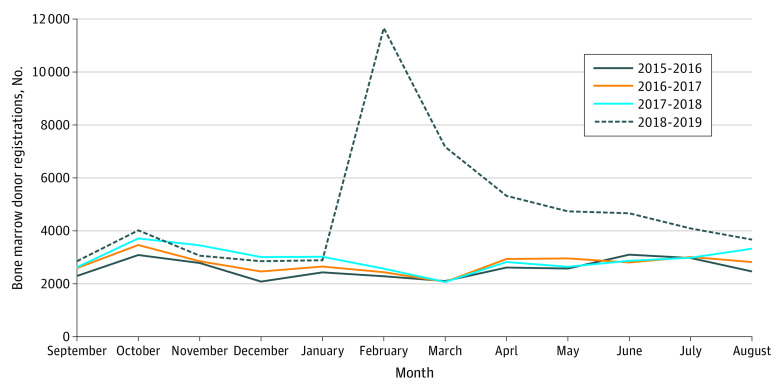
Trends in the Number of Bone Marrow Donor Registrations The dotted line denotes the intervention year (2018-2019) in which Ms Ikee disclosed her leukemia diagnosis in February 2019.

**Table.** Percentage Change in BMDRs Between the Intervention Time and the Control Time^a^

Month	Change, % (95% CI)	*P* value
September	6 (−2 to 15)	.12
October	10 (1 to 19)	.03
November	−5 (−16 to 7)	.35
December	7 (−11 to 29)	.48
January	[Reference]	NA
February	348 (318 to 379)	<.001
March	221 (183 to 265)	<.001
April	78 (61 to 96)	<.001
May	62 (41 to 87)	<.001
June	49 (25 to 77)	<.001
July	27 (14 to 43)	<.001
August	20 (4 to 38)	.02

Abbreviations: BMDRs, bone marrow donor registrations; NA, not applicable.

^a^ The intervention year was 2018-2019, and the control period included 2015-2016, 2016-2017, and 2017-2018.

## Discussion

This study found that Ms Ikee’s disclosure of leukemia was associated with the increase in BMDR. Because celebrity announcements rapidly spread via mass media and social networking platforms, our findings highlight that messages from high-profile figures may rapidly and substantially influence individuals’ behavior regarding health care choices for which decision-making is sometimes difficult, such as BMDRs.

Our study has limitations. First, due to the lack of data, our study could not identify the exact mechanisms through which BMDRs increased. Also, our findings may depend on the level of the celebrity’s popularity and may not be generalizable to a similar event that takes place in other countries.
